# Restrictive Versus Liberal Fluid Regimen in Refractory Sepsis and Septic Shock: A Systematic Review and Meta-Analysis

**DOI:** 10.7759/cureus.47783

**Published:** 2023-10-27

**Authors:** Ahmed M Abdelbaky, Wael G Elmasry, Ahmed H. Awad

**Affiliations:** 1 Critical Care, Intensive Care Unit, Dubai Academic Health Corporation - Rashid Hospital, Dubai, ARE; 2 Anesthesiology, Intensive Care Unit, Dubai Academic Health Corporation - Rashid Hospital, Dubai, ARE

**Keywords:** restrictive fluid regimen, liberal fluid regimen, septic shock, sepsis, fluid regimen

## Abstract

The optimal fluid management strategy for patients with sepsis remains a topic of debate. This meta-analysis aims to evaluate the impact of restrictive versus liberal fluid regimens on mortality, adverse events, and other clinical outcomes in patients with sepsis. We systematically reviewed 11 randomized controlled trials published between 2008 and 2023, comprising a total of 4,121 participants. The studies assessed 90-day mortality, 30-day mortality, adverse events, hospital length of stay, ICU admission rate, mechanical ventilation, ventilator-free days, ICU-free days, and vasopressor-free days. Quality assessments indicated minimal bias across the studies. The meta-analysis showed no statistically significant difference in 90-day mortality between restrictive and liberal fluid regimens (OR, 0.93; 95% CI, 0.80 to 1.70; P=0.30). Similar results were observed for 30-day mortality (OR, 0.73; 95% CI, 0.30 to 1.80; P=0.50). Adverse events were comparable between the two groups (OR, 0.81; 95% CI, 0.55 to 1.19; P=0.28). Furthermore, there were no significant differences in hospital length of stay (OR, 0.47; 95% CI, -0.85 to 1.80; P=0.48) or ICU admission rate (OR, 1.09; 95% CI, 0.66 to 1.77; P=0.75) between the restrictive and liberal fluid regimens. Regarding mechanical ventilation and ventilator-free days, no significant distinctions were observed (OR, 0.87; 95% CI, 0.65 to 1.17; P=0.48; OR, 0.99; 95% CI, -0.17 to 2.15; P=0.09, respectively). ICU-free days and vasopressor-free days also showed no significant differences between the two groups (OR, 0.97; 95% CI, -0.28 to 2.21; P=0.13; OR, -0.38; 95% CI, -1.14 to 0.37; P=0.32, respectively). This comprehensive meta-analysis of clinical trials suggests that restrictive and liberal fluid management strategies have comparable outcomes in patients with sepsis, including mortality, adverse events, and various clinical parameters. However, most studies favored restrictive fluid regimen over liberal approach regarding the number of vasopressor-free days, need for mechanical ventilation, adverse events, 30-day mortality, and 90-day mortality in sepsis patients.

## Introduction and background

Sepsis and septic shock remain critical challenges in modern medicine, contributing significantly to morbidity, mortality, and healthcare costs worldwide [1]. Sepsis is characterized as a potentially fatal organ dysfunction primarily caused by a faulty immune response. Septic shock represents the most severe form of sepsis [2]. In this sub-type, circulatory and cellular abnormalities are quite severe, which increase the risk of mortality. Furthermore, septic shock is characterized by profound hypotension and inadequate tissue perfusion despite adequate fluid resuscitation. Although there has been variability in reported mortality, estimates suggest that mortality due to sepsis is ≥10%, which can increase to ≥40% when septic shock is present [3,4].

Despite advances in medical science, the management of sepsis remains a complex puzzle with numerous therapeutic interventions under scrutiny [5]. Among these, fluid resuscitation strategies play a crucial part in the early phase of sepsis management. The purpose of intravenous fluid resuscitation is to optimize tissue perfusion and hemodynamic stability. In sepsis, there is a reduction in intravascular volume due to vasodilated vascular network [6]. Therefore, intravenous fluid augments the macrovascular perfusion (e.g., stroke volume and cardiac output) and microvascular perfusion (e.g., capillary blood flow). Generally, early and aggressive fluid administration has been a cornerstone of sepsis management for decades. However, aggressive fluid resuscitation is linked to fluid overload, edema, and dilutional coagulopathy [7]. Apart from fluid resuscitation, clinicians also use vasopressors to reduce hypoperfusion caused by sepsis [8]. The common practice includes a combination of both therapies for sepsis-induced hypoperfusion.

Historically, the liberal approach to fluid resuscitation advocated for the administration of generous amounts of intravenous fluids, aiming to rapidly correct hypovolemia and improve cardiac output [9]. This approach was based on the assumption that aggressive fluid administration would enhance tissue perfusion, mitigate the risk of organ failure, and reduce mortality. However, the unintended consequences of fluid overload, such as pulmonary edema, abdominal compartment syndrome, and impaired oxygen exchange, have sparked a reevaluation of this strategy [10]. A recent research by Della Rocca et al. has questioned the conventional wisdom of liberal fluid administration in sepsis, as accumulating evidence suggests potential harm associated with fluid overload [11].

In contrast, the restrictive fluid resuscitation approach emerged from concerns regarding the potential harms associated with fluid overload [12]. This approach emphasizes careful monitoring and cautious administration of fluids, with the goal of preventing iatrogenic complications and maintaining a state of euvolemia rather than inducing supra-physiological levels of intravascular volume. Proponents of the restrictive strategy argue that limiting fluid administration can mitigate the risk of organ dysfunction, decrease the need for invasive interventions such as mechanical ventilation, and ultimately lead to improved patient outcomes [13]. So far, a significant body of evidence has emerged regarding the efficacy of both fluid resuscitation approaches in sepsis and septic shock patients. Multiple randomized controlled trials (RCTs) and observational studies have explored the clinical implications of liberal versus restrictive fluid strategies [14,15], yielding conflicting results. This meta-analysis seeks to comprehensively evaluate the existing evidence, elucidate the underlying mechanisms driving the effects of fluid resuscitation, and offer a nuanced understanding of when and how these strategies might be best employed in the management of refractory sepsis and septic shock.

## Review

Methodology

This meta-analysis was conducted in accordance with the guidelines outlined in the Cochrane Handbook for Systematic Reviews of Interventions [16]. The predetermined protocol was duly registered in the International Prospective Register of Systematic Reviews (PROSPERO) with the registration number CRD42023461930. For this meta-analysis, all RCTs that were published up through 2023 were considered. The present meta-analysis has been conducted in adherence to the Preferred Reporting Items for Systematic Reviews and Meta-Analyses (PRISMA) guidelines [17]. Given that the individual studies included in the meta-analysis had already obtained ethical approval, there was no need for additional permission for this systemic review. The PICOS protocols for this meta-analysis were as follows: (P) patients with sepsis or septic shock; the intervention (I) being the use of liberal or restrictive fluid regimen; the control (C) patients on either restrictive or liberal fluid regimen; and the outcomes (O) 90-day mortality, vasopressor-free days, adverse events, ICU-free days, and hospital length of stay.

Search Strategy and Data Sources

For this systemic review and meta-analysis, we performed a comprehensive search in various databases including PubMed, CINAHL Ultimate, Web of Science, and Scopus to identify relevant studies that evaluated liberal versus restrictive fluid regimen in sepsis. The search was carried out by using a combination of various keywords including “hemodynamic support,” or “fluid resuscitation,” or “liberal versus restrictive fluid strategy,” and “sepsis,” or “septic shock.” Furthermore, related terms and alternatives to these keywords were also used for the systemic search. The details of keywords and different Boolean operators used in search are mentioned in the Appendix. To identify more studies related to the topic, a search was also undertaken in Google Scholar. The inclusion criteria of the studies comprised the following: (1) compared the liberal or restrictive fluid regimen in sepsis and septic shock patients, (2) reported clinical outcomes in patients, (3) studies were published in English language. Only patients with sepsis or septic shock were considered for this meta-analysis. Patients who underwent liberal or restrictive fluid regimen but were not suffering from sepsis or septic shock were excluded.

Data Extraction

After searching various databases, the results were exported to an Endnote file. All the retrieved files from the database search were transferred to Endnote, the reference manager. Then, all duplicates were removed in Endnote. The remaining results were uploaded to Rayyan, a web-based software for conducting systemic reviews [18]. At this stage, two independent reviewers were involved in the study selection process. Both reviewers were blinded during the selection process by turning on the “blind” option in Rayyan. In the first step, the authors made a decision based on the title and abstract of the records. In the second step, the blind was removed, and the decision of inclusion or exclusion was compared until a shared decision was reached. In case of any disagreements, a third reviewer was involved to make the final decision. Finally, data were obtained regarding demographics, study design, intervention used, and outcomes of the study.

Risk-of-Bias Assessment

The risk-of-bias assessment tool in Review Manager (RevMan) version 5.3 software, developed by The Cochrane Collaboration in Oxford, UK, in 2014, was used by two authors to independently evaluate the risk of bias. The study's evaluation encompassed several key factors: the presence of proper sequence generation to minimize selection bias, the concealment of allocation to prevent selection bias, the prevention of knowledge regarding assigned interventions during the study to minimize performance bias, the appropriate blinding of participants and personnel to minimize performance bias, the blinding of outcome assessors to minimize detection bias, the adequate handling of incomplete outcome data to minimize attrition bias, the absence of selective outcome reporting in the study's report to minimize reporting bias, and the absence of other potential issues that could introduce bias and jeopardize the study's validity. All issues were effectively settled by the third author.

Outcome Measures

The primary outcome was 90-day mortality in participants. The secondary outcomes included adverse events, hospital length of stay, ICU admission rate, ICU-free days, mechanical ventilation, ventilator-free days, and vasopressor-free days.

Data Analysis

A random-effects meta-analysis was conducted using the DerSimonian and Laird method [19]. This approach accounts for potential heterogeneity across studies and provides a pooled estimate of the effect size along with a 95% confidence interval (CI). The effect size used in the analysis was the odds ratio (OR) for binary outcomes. The assessment of heterogeneity among the included studies was conducted using the I² statistic, which measures the proportion of overall variation across studies that can be attributed to heterogeneity rather than random chance. A value of I² greater than 50% indicates substantial heterogeneity. The results of the meta-analysis are reported using forest plots, displaying the pooled effect sizes and their corresponding 95% CIs. The statistical significance of the results is indicated by p-values, with a threshold of 0.05.

Results

Included Studies

A total of 8,462 articles were generated from the databases (1,765 articles from PubMed, 3,403 from Scopus, 2,488 from Web of Science, and 806 from CINAHL Ultimate). Furthermore, 106 articles were also identified from Google Scholar. After removal of duplicates, 7,873 remained articles, which were further excluded based on the title and abstract of the articles, leaving 1,023 articles for further analysis. Finally, after exclusion of all non-relevant articles by independent reviewers, only 11 articles were included for final analysis.

Flow Diagram

**Figure 1:**
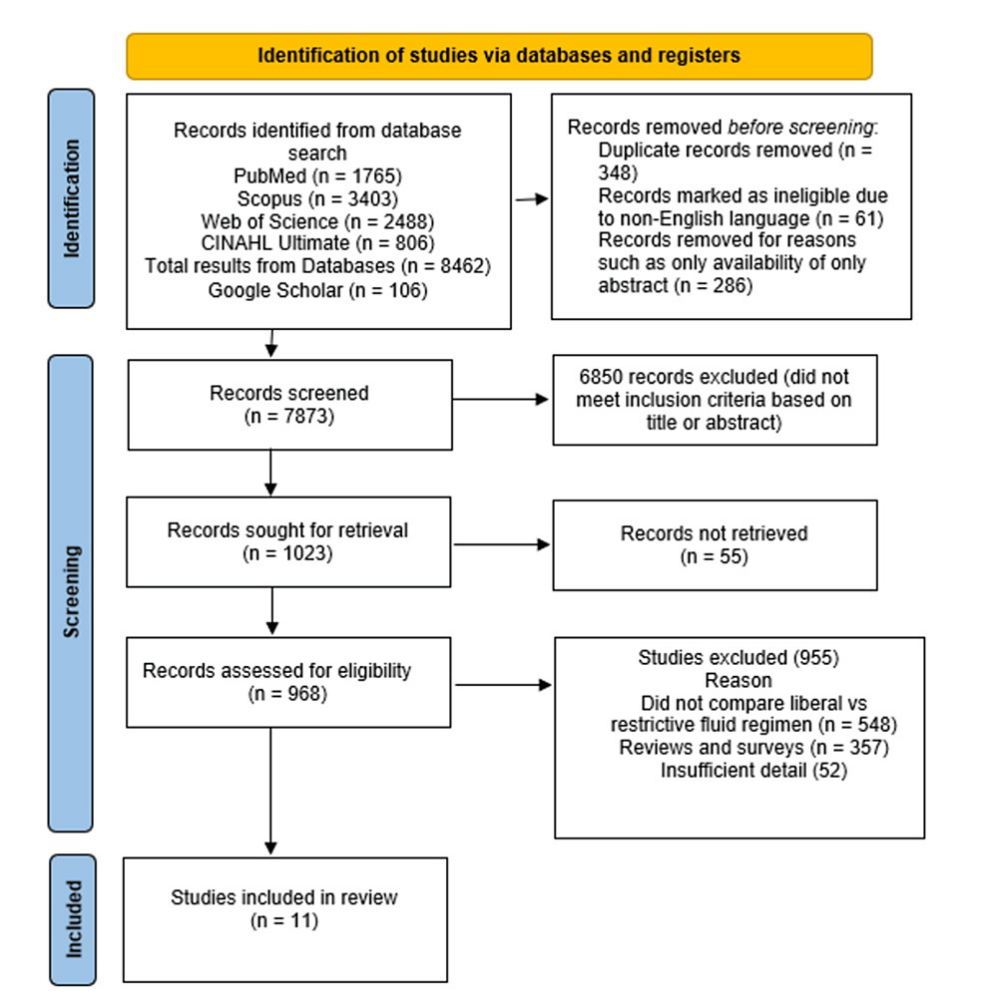
Flow diagram of the systemic review and meta-analysis

Figure 1 shows the PRISMA flow diagram of the systemic review and meta-analysis.

Characteristics of Included Studies

A total of 11 RCTs were included in the meta-analysis including a total of 4,121 participants (Table 1). Regarding outcome measures, the cumulative 90-day mortality was 28.36% (558/1967) in the restrictive fluid regimen group and 29.93% (594/1984) in the liberal fluid regimen group in nine studies. Two studies did not report 90-day mortality [20,21].

**Table 1:** Characteristics of studies included in the systematic review and meta-analysis ICU, intensive care unit; PRR, protocol restricting resuscitation

Author	Year	Participants			Follow-up period	Complications/adverse events	Conclusion
		Total	Restrictive	Liberal			
Shapiro et al. [22]	2023	1563	782	781	90-day follow-up	Higher deaths were reported in the liberal group compared to the restrictive group (116 vs 109; P=0.61).	No significant difference was found in mortality outcomes.
Meyhoff et al. [23]	2022	1554	1554	784	90-day follow-up	Adverse events occur in 221 out of 751 in the restrictive group and 238 out of 772 in the liberal group.	The intravenous fluid restriction was found to cause no decrease in the death rate at 90 days when compared to the standard intravenous fluid therapy
Corl et al. [20]	2020	109	55	54	30 days	12 deaths were reported in the restrictive group, while 12 deaths were reported in the liberal group. There were no significant differences in adverse events in both groups.	The implementation of a restricted resuscitation strategy showed a beneficial impact on the administration of intravenous fluids in patients diagnosed with severe sepsis and septic shock, as compared to the standard care group consisting of sepsis patients.
Semler et al. [24]	2019	30	15	15	N/A	No mortality was reported. There was no difference in adverse events.	The results of the phase II trial indicate that the implementation of a conservative fluid management plan did not result in a statistically significant reduction in the average daily fluid balance exceeding 500 mL in patients diagnosed with sepsis.
Inwald et al. [21]	2019	73	39	34	30-day follow-up	No mortality was reported. There was no significant difference in adverse events among the two groups.	Participants were found to be not as unwell as they were expected to be.
Macdonald et al. [25]	2018	99	50	49	90-day follow-up	Mortality was reported in four patients in the restrictive and three in the liberal group. Adverse effects were reported in eight patients in both groups.	The use of a protocol involving limited fluid intake and prompt administration of vasopressor medication in emergency department patients presenting with indications of sepsis and low blood pressure seems to be a viable approach.
Andrews et al. [26]	2017	212	107	105	28-day follow-up	Mortality was reported in 51/106 and 34/103 patients in restrictive and liberal groups, respectively. The adverse effects rate was similar in both groups.	The intervention group received more vasopressors and fluids.
Hjortrup et al. [27]	2016	151	75	76	90-day follow-up	Mortality was reported in 25/75 in the restrictive group and 31/76 in the liberal group; p = 0.32). Adverse effects occurred in 30 and 48 patients in the restrictive and liberal groups, respectively.	In adult patients with septic shock in ICU, the implementation of a PRR fluid showed efficacy in reducing the quantities of resuscitation fluid as compared to a standard care strategy.
Chen and Kollef [28]	2015	82	41	41	5-day follow-up	Mortality was reported in 56.1% and 48.8% of patients in the restrictive and liberal groups, respectively. There was no difference in the adverse effects.	Fluid minimization in patients with septic shock can be performed by the use of fluid responsiveness protocol assessments. However, larger trials are needed in septic shock.
Benakatti et al. [29]	2012	101	N/A	N/A	12 months Follow-up	An insignificant mortality rate was reported in both groups (18.55 and 23.45, respectively).	A restrictive fluid treatment has improved the function of the lungs such as shortening the ventilation and stay in ICU without provoking the instability of hemodynamics
Santhanam et al. [30]	2008	147	74	73	1-hour follow-up	Mortality was recorded in 26 patients 13 in each group. Adverse effects were not significantly different.	Insignificant difference was reported in whole mortality, incidence of complications among the two groups, and shock resolution speediness

Quality Assessment of the Included Studies

**Figure 2:**
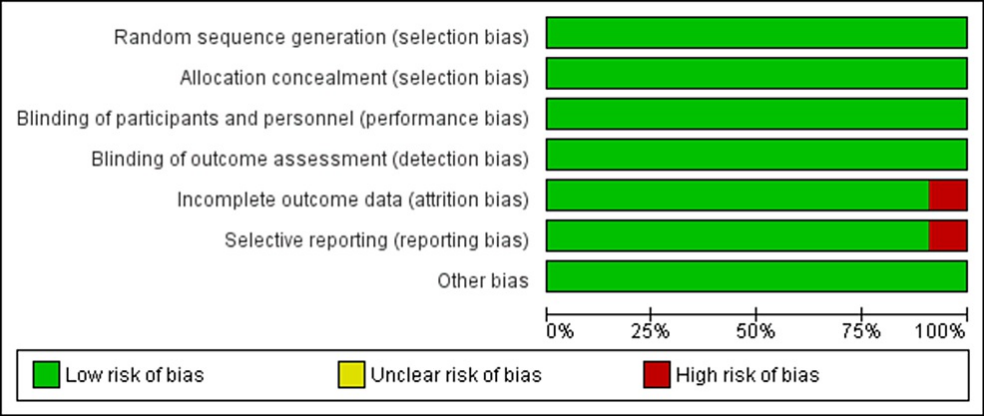
Risk-of-bias graph of all included studies presenting the review authors’ judgments about each risk-of-bias item

**Figure 3:**
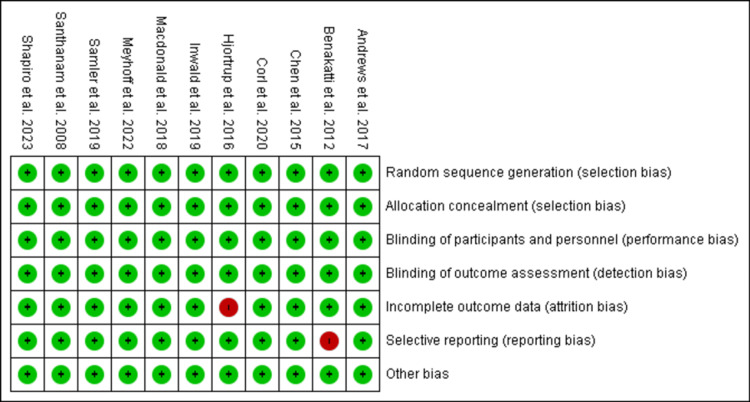
Risk-of-bias summary for each of the study Andrews et al. [26], Benakatti et al. [29], Chen and Kollef [28], Corl et al. [20], Hjortrup et al. [27], Inwald et al. [21], Macdonald et al. [25], Meyhoff et al. [23], Semler et al. [24], Santhanam et al. [30], Shapiro et al. [22]

The risk-of-bias assessments for the 11 papers included in the meta-analysis are detailed in Figures 2, 3. All 11 (100%) studies were assessed to have a minimal risk of bias in terms of random sequence creation, attrition bias, and reporting bias. Furthermore, all studies were determined to have a low risk of allocation concealment bias, which is a form of selection bias. Similarly, all studies were assessed to have minimal risk of bias in terms of performance bias (blinding of participants and investigators) and detection bias (blinding of outcome assessors). A total of 10 (91%) studies had minimal bias regarding incomplete outcome data. Reporting bias was reported in only one study included in the systemic review. No study had other biases.

Mortality Outcomes

**Figure 4:**
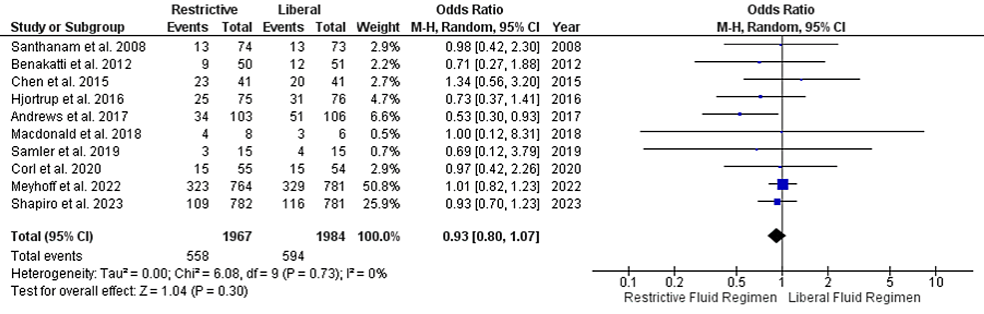
The 90-day mortality outcome Santhanam et al. [30], Benakatti et al. [29], Chen and Kollef [28], Hjortrup et al. [27], Andrews et al. [26], Macdonald et al. [25], Semler et al. [24], Corl et al. [20], Meyhoff et al. [23], Shapiro et al. [22] SD, standard deviation; CI, confidence interval; M-H, Mantel-Haenszel

**Figure 5:**
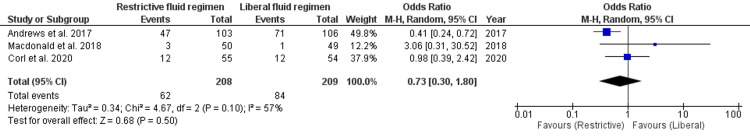
The 30-day mortality outcome Andrews et al. [26], Macdonald et al. [25], Corl et al. [20] SD, standard deviation; CI, confidence interval; M-H, Mantel-Haenszel

A total of nine studies involving 3,939 participants reported 90-day mortality outcomes and were included in the analysis. The 90-day mortality difference between restrictive and liberal fluid regimens was not statistically significant (OR, 0.93; 95% CI, 0.80 to 1.70; P=0.30; I^2^=0% [low heterogeneity]) (Figure 4). Data from three studies was analyzed for 30-day mortality. Similarly, there was no significant difference regarding 30-day mortality in restrictive and liberal fluid regimens (OR, 0.73; 95% CI, 0.30 to 1.80; P=0.50; I^2^=57% [moderate heterogeneity]) (Figure 5).

Adverse Events

**Figure 6:**
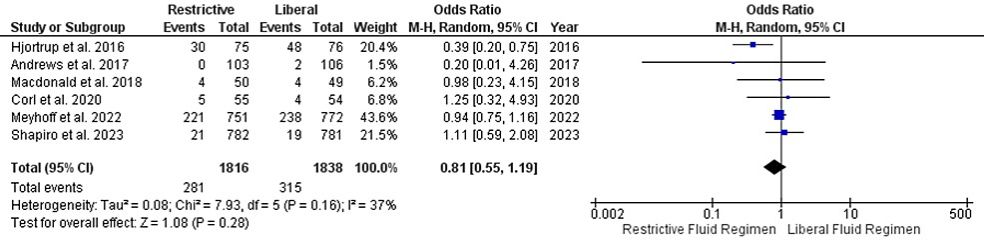
Adverse events reported in the included studies Hjortrup et al. [27], Andrews et al. [26], Macdonald et al. [25], Corl et al. [20], Meyhoff et al. [23], Shapiro et al. [22] SD, standard deviation; CI, confidence interval; M-H, Mantel-Haenszel

A total of six studies reported adverse outcomes and were included in the analysis. Adverse events in both restrictive and liberal fluid regimens were comparable (OR, 0.81; 95% CI, 0.55 to 1.19; P=0.28). The I^2^ value for the analysis was 37%, which indicates moderate heterogeneity (Figure 6).

Hospital Length of Stay and ICU Admission Rate

**Figure 7:**
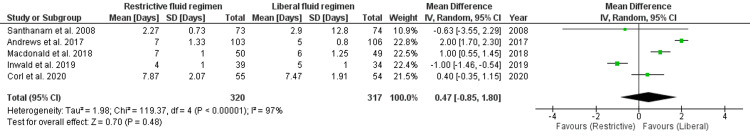
Hospital length of stay Santhanam et al. [30], Andrews et al. [26], Macdonald et al. [25], Inwald et al. [21], Corl et al. [20] SD, standard deviation; CI, confidence interval; M-H, Mantel-Haenszel

**Figure 8:**
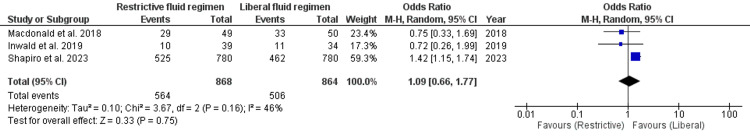
ICU admission rate Macdonald et al. [25], Inwald et al. [21], Shapiro et al. [22] SD, standard deviation; CI, confidence interval; M-H, Mantel-Haenszel; ICU, intensive care unit

Five studies were included in the analysis that reported hospital length of stay. In both restrictive and liberal fluid groups, there was no significant difference in hospital length of stay (OR, 0.47; 95% CI, -0.85 to 1.80; P=0.48). The I^2^ value for the analysis was 97%, which indicates considerable heterogeneity (Figure 7). Only three studies reported ICU admission rate, which were included in the analysis. ICU admission rates in both restrictive and liberal fluid regimens were comparable (OR, 1.09; 95% CI, 0.66 to 1.77; P=0.75). The I^2^ value for the analysis was 46%, which indicates moderate heterogeneity (Figure 8).

Other Outcome Measures

**Figure 9:**
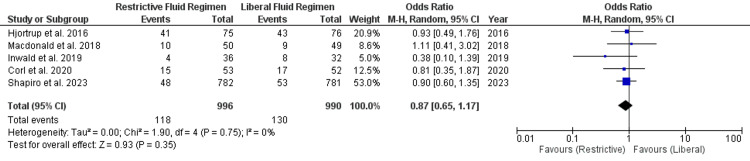
Forest plot of mechanical ventilation used Hjortrup et al. [27], Macdonald et al. [25], Inwald et al. [21], Corl et al. [20], Shapiro et al. [22] SD, standard deviation; CI, confidence interval; M-H, Mantel-Haenszel

**Figure 10:**
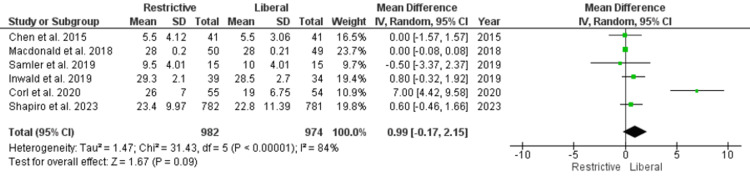
Ventilator-free days Chen and Kollef [28], Macdonald et al. [25], Semler et al. [24], Inwald et al. [21], Corl et al. [20], Shapiro et al. [22] SD, standard deviation; CI, confidence interval; M-H, Mantel-Haenszel

**Figure 11:**
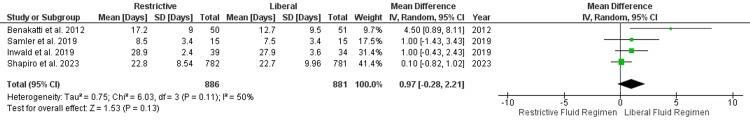
ICU-free days Benakatti et al. [29], Semler et al. [24], Inwald et al. [21], Shapiro et al. [22] SD, standard deviation; CI, confidence interval; M-H, Mantel-Haenszel; ICU, intensive care unit

**Figure 12:**
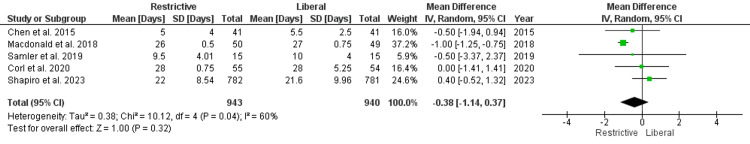
Vasopressor-free days Chen and Kollef [28], Macdonald et al. [25], Semler et al. [24], Corl et al. [20], Shapiro et al. [22] SD, standard deviation; CI, confidence interval; M-H, Mantel-Haenszel

Five studies were included in the analysis that reported mechanical ventilation. In both restrictive and liberal fluid groups, there was no significant difference in mechanical ventilation (OR, 0.87; 95% CI, 0.65 to 1.17; P=0.48). The I^2^ value for the analysis was 0%, which indicates no heterogeneity (Figure 9). Only six studies reported ventilator-free days, which were included in the analysis. Number of ventilator-free days in both restrictive and liberal fluid regimens were comparable (OR, 0.99; 95% CI, -0.17 to 2.15; P=0.09). The I^2^ value for the analysis was 84%, which indicates considerable heterogeneity (Figure 10). Number of ICU-free days in both restrictive and liberal fluid regimens were comparable (OR, 0.97; 95% CI, -0.28 to 2.21; P=0.13). The I^2^ value for the analysis was 50%, which indicates moderate heterogeneity (Figure 11). Number of ICU-free days in both restrictive and liberal fluid regimens were comparable (OR, -0.38; 95% CI, -1.14 to 0.37; P=0.32). The I^2^ value for the analysis was 60%, which indicates moderate heterogeneity (Figure 12).

Discussion

Sepsis is a complex and life-threatening condition that requires careful management, and intravenous fluid therapy is a crucial component of its treatment [9]. This meta-analysis aimed to evaluate the impact of restrictive versus liberal fluid regimens in patients with sepsis. The key outcomes focused were mortality, adverse events, hospital length of stay, ICU admission rate, and other relevant measures. The findings of this meta-analysis shed light on the ongoing debate regarding the optimal fluid management strategy for sepsis patients. One of the primary concerns in sepsis management is mortality reduction. The analysis of 90-day mortality outcomes from nine studies did not reveal a statistically significant difference between restrictive and liberal fluid regimens. However, most of the studies included in the analysis showed lower mortality in the restrictive group compared to the liberal group [22-24,26,27,29]. Only two studies showed higher mortality in the liberal group compared to the restrictive group [25,28]. However, both of these studies had a small sample size, and the difference in outcome measures was not statistically significant. Only one RCT by Corl et al. reported 60-day mortality; therefore, there was not enough data for analysis. Their findings also showed no statistically significant difference in mortality outcomes in the liberal or conservative group [20]. The findings suggest that neither approach confers a substantial advantage in terms of reducing mortality. The lack of significant heterogeneity in this outcome (I^2^=0%) enhances the robustness of this conclusion.

It is noteworthy that the results regarding 30-day mortality were also consistent with those of 90-day mortality. This suggests that the short-term effects of fluid management strategies do not significantly impact patient survival. These findings challenge the conventional belief that a more liberal fluid approach might improve outcomes by ensuring adequate tissue perfusion. Another critical aspect of fluid management is the occurrence of adverse events. Our analysis revealed that adverse events were comparable between restrictive and liberal fluid regimens. This finding aligns with the observations of several studies, including those by Andrews et al. [26], Macdonald et al. [25], and Corl et al. [20]. These studies reported similar rates of adverse events in both groups, suggesting that a more conservative fluid strategy does not necessarily lead to a higher incidence of complications. However, there was moderate heterogeneity in this outcome (I^2^=37%), suggesting some variability in the reported adverse events across studies. The absence of a significant difference in adverse events between the two approaches is reassuring, as it indicates that a restrictive strategy does not compromise patient safety. Given the potential risks associated with fluid overload, the adoption of a more conservative fluid strategy appears justifiable.

Hospital length of stay and ICU admission rate are essential components of the healthcare burden associated with sepsis. Our analysis found no significant difference in hospital length of stay between restrictive and liberal fluid regimens. However, there was substantial heterogeneity in this outcome (I^2^=97%). This variability in reported hospital lengths of stay across studies may reflect differences in patient populations, treatment protocols, or healthcare systems. Similarly, ICU admission rates did not significantly differ between the two fluid management strategies, with moderate heterogeneity in this outcome (I^2^=46%). These findings suggest that fluid strategy alone does not strongly influence the duration of hospitalization or the likelihood of ICU admission in sepsis patients. It is important to note that hospital length of stay and ICU admission rate are influenced by a multitude of factors beyond fluid management, such as comorbidities, severity of illness, and local healthcare practices. Therefore, while our analysis did not identify a significant impact of fluid strategy on these outcomes, clinicians should consider the broader clinical context when making decisions regarding fluid resuscitation.

Additional outcome measures included mechanical ventilation, ventilator-free days, and ICU-free days. Our analysis did not find a significant difference in mechanical ventilation between restrictive and liberal fluid regimens, and there was no heterogeneity in this outcome (I^2^=0%). Similarly, the number of ventilator-free days did not significantly differ between the two strategies, but there was considerable heterogeneity (I^2^=84%). However, the p-value for this parameter was close to statistical significance (P=0.09). Most studies included in the analysis showed that the restrictive fluid group had more ventilator-free days compared to the liberal fluid approach [20-22]. ICU-free days and vasopressor-free days also showed no significant differences between the two fluid management approaches, with moderate heterogeneity (I^2^=50% and I^2^=60%, respectively). These findings suggest that restrictive fluid strategies do not compromise patients' respiratory or hemodynamic status when compared to more liberal approaches. The heterogeneity observed in some of these outcomes may be attributed to variations in patient populations, clinical practices, and study methodologies across the included studies. It underscores the need for caution in interpreting these results and highlights the importance of considering individual patient characteristics when determining the appropriate fluid strategy.

Limitations

Although the present meta-analysis provides significant evidence regarding the liberal versus restrictive fluid regimen, there are several limitations. First, the analysis demonstrated a lack of consistent statistical significance across certain outcomes. For instance, the observed reduction in mortality among patients receiving restrictive fluid did not reach statistical significance. This could be attributed to the relatively small effect sizes, the variation in study methodologies, or the limitations of the available data. The clinical implications of these findings might be less straightforward than implied by the reported ORs. Second, despite efforts to minimize bias, the risk of publication bias cannot be entirely ruled out. Additionally, the follow-up periods of the included studies varied, which may influence the detection of longer-term outcomes. Finally, the results of this meta-analysis should be considered in conjunction with the clinical context and the condition of patients.

## Conclusions

In conclusion, this meta-analysis of 11 studies involving more than 4,000 sepsis patients suggests that restrictive and liberal fluid regimens yield comparable outcomes in terms of mortality, adverse events, hospital length of stay, ICU admission rate, and other relevant measures. However, most studies included in the analysis favored a restrictive approach of fluid management. Clinicians should carefully consider the individual patient's clinical status, comorbidities, and specific clinical context when making decisions regarding fluid resuscitation. Further research, including large-scale RCTs, is needed to refine our understanding of optimal fluid management strategies in sepsis and to identify subpopulations that may benefit from specific approaches.

## Appendices

**Table 2:** Terms and strategy used for literature search

Terms and strategy
#1 Restrictive fluid strategy
#2 Liberal fluid strategy
#3 Liberal fluid regimen
#4 Restrictive fluid regimen
#5 Liberal versus restrictive fluid strategy
#6 Liberal vs restrictive hemodynamic support
#7 Hemodynamic support
#8 Fluid resuscitation
#9 Intravenous fluid management
#10 OR/1-9
#11 Sepsis
#12 Septic shock
#13 Refractory sepsis
#14 Septic infection
#15 OR/11-14
#16 10 AND 15
